# Increased Insular Connectivity and Enhanced Empathic Ability Associated with Dance/Music Training

**DOI:** 10.1155/2019/9693109

**Published:** 2019-05-06

**Authors:** Li Gujing, He Hui, Li Xin, Zhang Lirong, Yao Yutong, Ye Guofeng, Lu Jing, Zhou Shulin, Yang Lei, Luo Cheng, Yao Dezhong

**Affiliations:** ^1^The Clinical Hospital of Chengdu Brain Science Institute, MOE Key Lab for Neuroinformation, Center for Information in Medicine, School of Life Science and Technology, University of Electronic Science and Technology of China, Chengdu 610054, China; ^2^Art and Culture Centre, University of Electronic Science and Technology of China, Chengdu, China; ^3^Faculty of Natural Sciences, University of Stirling, Stirling, UK; ^4^School of Music Education, Sichuan Music Conservatory, Chengdu, China; ^5^School of Drama, Sichuan Music Conservatory, Chengdu, China

## Abstract

Dance and music are expressive art forms. Previous behavioural studies have reported that dancers/musicians show a better sensorimotor ability and emotional representation of others. However, the neural mechanism behind this phenomenon is not completely understood. Recently, intensive researches have identified that the insula is highly enrolled in the empathic process. Thus, to expand the knowledge of insular function associated with empathy under the dance/music training background, we mapped the insular network and its associated brain regions in 21 dancers, 20 musicians, and 24 healthy controls using resting-state functional connectivity (FC) analysis. Whole brain voxel-based analysis was performed using seeds from the posterior insula (PI), the ventral anterior insula (vAI), and the dorsal anterior insula (dAI). The training effects of dance and music on insular subnetworks were then evaluated using one-way analysis of variance ANOVA. Increased insular FC with those seeds was found in dancers/musicians, including PI and anterior cingulated cortex (ACC), vAI and middle temporal gyrus (MTG) and middle cingulated cortex (MCC), and dAI and ACC and MTG. In addition, significant associations were found between discrepant insular FC patterns and empathy scores in dancers and musicians. These results indicated that dance/music training might enhance insular subnetwork function, which would facilitate integration of intero/exteroceptive information and result in better affective sensitivity. Those changes might finally facilitate the subjects' empathic ability.

## 1. Introduction

Dance and music are expressive art forms. Based on high levels of integration of external/internal sensorimotor stimuli, dancers/musicians can make perfect performance and express emotions through sequences of tones or series of choreographic elements. Dance training is related to whole-body control, spatial integration, rhythmicity synchronization, action observation, and imitation [1]^.^ Music training is related to the control of delicate hand or finger movements with rapid feedback of auditory, visual, and motor interaction [2]. At present, researches on dance training-induced plasticity are mainly on “action observation network” (AON) [3]. This network is part of the mirror neuron system (MNS) and sensitive to whole-body action observation and movement learning which is highly related in the sensorimotor process [4, 5]. Compared with dance, studies on music training-induced plasticity are massive and more systematic, including the auditory system [6–8], motor network [9, 10], multimodal interactions and integration in musical training [11, 12] and the reward system [13, 14]. Besides the basic neuroplasticity findings, others have reported the transfer effects induced by dance/music training. The transfer effects means the transferability of training-related benefits to new or unfamiliar situations [15]. For example, Rehfeld and his colleagues found that an 18-month dance training induced an increased hippocampal volume and higher balance function [16]. Another study reported that language processing and dance watching might share certain neural mechanisms and dance training might affect the syntax ability [17]. Fink and his colleagues discovered stronger alpha synchronization in dancers during high creative thinking compared with controls [18]. Similar results were reported in music researches as well. Studies found music transfer effects with language pitch [19, 20], semantic ability [21], verbal memory [22, 23], executive function [24], attention [25], emotional perception [26], auditory skills [27], and so on.

Empathy is a complex psychological response, which forms insights into the thoughts and feelings of others from within their frame of reference [28]. The shared representations are one of the neurophysiological bases for empathy, which are associated with the perception and execution of actions. Perception of a given behaviour in another individual automatically activates one's own representations of that behaviour and further helps one to form the similar feelings and thoughts of others [29]. As mentioned earlier, dance/music activities include a massive sensorimotor process related to action execution and observation, which constantly recruit the sensorimotor cortex and action observation network. Would this process facilitate the empathic ability? Are there shared fundamental neural mechanisms that could serve as the basis for dance/music expertise and empathic ability? Recently, the insula has received intensive attention. Researchers have reported that this structure was closely attended in the empathy process [30]. The insula has been identified as a hub of saliency network (SN) and facilitates the generation of subjective feelings and emotions from the integration of interoceptive and exteroceptive sensory information [31, 32]. It is functionally distinguished into the sensorimotor posterior insula (PI), the affective ventral anterior insula (vAI), and the cognitive dorsal anterior insula (dAI) which integrates the interactions between other brain networks and involves in externally oriented attention and full representation of self [32, 33]. Lines of studies have further indicated overlapping patterns of insular activities when someone experiences an subjective and vicarious emotional state [30, 34]. For instance, many literatures reported that the anterior insula, MCC, and ACC were the core structure responses to empathy for pain [30, 35]. Other studies worked on empathy for vicarious emotions and sensations, including anxiety [36], social exclusion [37], disgust [38], and taste [39]. They have indicated that the dACC-aMCC-SMA and bilateral anterior insula supported a core function underlying empathy [40].

Resting-state functional magnetic resonance imaging (rs-fMRI) is based on the association of intrinsic spontaneous low-frequency blood oxygenation level-dependent (BOLD) signal fluctuations between spatially remote brain regions. Relevant research has reported that rs-fMRI might be closely related to neural subsystems revealed by task-activation fMRI [41]. Our previous study has used rs-fMRI to demonstrate altered brain functional connectivity (FC) of dancers in sensorimotor networks induced by dance training [42]. Furthermore, we applied the independent component analysis (ICA) to analyse the altered functional network connectivity in musicians [43]. Therefore, in this study we continuously used rs-fMRI to map interregional FC and compared connectivity patterns among dancers, musicians, and healthy controls related to the insula.

Previous literature suggests that long-term dance/music training could affect brain plasticity and induce transfer effects. The perception and execution of actions, which are common in dance/music training, are related to shared representation which is important for empathy. According to this possible link, this study is aimed at investigating the shared fundamental neural mechanisms and tried to explain the transfer effects of art training on empathic ability. We intensively focused on insula, as its important role in sensorimotor representation and self-agency which are crucial for empathy. We hypothesized that the dance/music training might enhance insular subnetwork function and would have a positive effect on empathic ability. As such, we designed a cross-sectional experiment (dancers, musicians, and healthy controls) and used rs-FC to test our hypothesis. The analysis of rs-FC was performed to assess functional differences in insular networks induced by dance/music training. Then, we made further investigation on the association between FC and individual empathy scores in order to ascertain possible neuro-evidence for the transfer effects under dance/music training.

## 2. Materials and Methods

### 2.1. Subjects and Study Design

Two groups of subjects were recruited for this study, including 23 dancers (dancer group) and 23 musicians (musician group). A set of rs-fMRI data from 24 healthy controls (control group) were obtained from the University of Electronic Science and Technology of China (UESTC) Imaging Center. The participants in the expert groups consisted of college students majoring in modern dance and western string instrument from the Southwest Minzu University and UESTC, respectively. All subjects were assessed by a group of senior dancers and musicians. Subjects, who were qualified according to the admission requirements for a professional modern dance student or western string music student, were defined as dancers or musicians. Specifically, dancers were assessed in three tasks. (1) The flexibility task includes straddle and front splits, bend backward and forward, etc. (2) In the imitation task, a series of dance movements lasting 60 seconds which were unfamiliar to the dancers were shown three times. Then, the dancers were required to recall the movements and try to perform the pieces of dance as completely as possible. (3) In the impromptu dance task, the dancers needed to create a dance according to a segment of random selected music. For musicians, they were assessed in two tasks: (1) in the selected music performance task, the musicians were required to play a prepared music about 3 minutes. (2) In the sight-reading task, the musicians needed to sight-read and play 16 bars of unfamiliar music without preparation. The dancers and musicians engaged in regular dance or music training, respectively, for an average of 12 hours per week over an extended period of time ranging from 7 to 17 years. We excluded subjects whose training periods were too irregular as determined by a questionnaire survey. All the subjects were right-handed according to the Edinburgh Inventory [44]. We used a questionnaire to assess in regards to past or current developmental disorders, alcohol or substance abuse, and neurological or psychiatric disorder. None of the participants had reported any of those. All imaging was performed at the UESTC Imaging Centre. The study was consistent with the Declaration of Helsinki [45] and approved by the UESTC Ethics Board. Written informed consent was obtained from each subject prior to the scanning.

### 2.2. Psychological Measurements

All the expert participants (dancers and musicians) completed the Chinese version of the Interpersonal Reactivity Index (C-IRI). This self-rating instrument is originated from the Interpersonal Reactivity Index (IRI) which has been commonly used in empathy studies and has shown a good psychometric quality in Chinese people [46]. It contains four subscales, including a perspective-taking scale (PT), fantasy scale (FS), empathic concern scale (EC), and personal distress scale (PD). The PT assesses the spontaneous attempts to adopt the perspectives of other people and see things from their point of view. The FS measures the tendency to identify with characters in movies, novels, plays, and other fictional situations. The EC assesses respondents' feelings of warmth, compassion, and concern for others, while the PD measures the personal feelings of anxiety and discomfort that result from observing another's negative experience. The PT concerns the cognitive components of empathy, and the other three measure the affective components of empathy. Because the fMRI data of healthy controls were obtained from the UESTC Imaging Centre, there were no corresponding C-IRI scores with the healthy controls from the database. To make a comparison, we used the mean scores of the C-IRI from a large Chinese sample (*n* = 529) as a reference [46].

### 2.3. Data Acquisition

The experiments were performed on a 3T MRI scanner (GE Discovery MR750) in the Centre for Information in Medicine of UESTC. High-resolution T1-weighted images were acquired using a 3-dimensional fast spoiled gradient echo sequence (repetition time [TR] = 6.008 ms, flip angle [FA] = 9, matrix = 256 × 256, field of view [FOV] = 256 × 256 mm^2^, slice thickness = 1 mm, no gap, and 152 slices). Subsequently, the gradient-echo echo-planar imaging (EPI) sequence was used to acquire the functional images. The main scan parameters were as follows: TR = 2000 msec, TE = 30 msec, FA = 90°, matrix = 64 × 64, FOV = 24 × 24 cm^2^, and slice thickness/gap = 4 mm/0.4 mm, with an eight-channel phased array head coil. All subjects underwent a 510-second resting-state scan to yield 255 volumes (32 slices per volume).

### 2.4. Data Analysis

Functional data preprocessing was performed using SPM8 (Statistical Parametric Mapping, https://www.fil.ion.ucl.ac.uk/spm/). Slice timing corrections, head motion corrections, normalization (3 *mm* ^∗^ 3 *mm* ^∗^ 3 *mm*) into the echo planar image template, and spatial smoothing (Gaussian kernel of a full width at half-maximum (FWHM) of 6 mm) were carried out. Sources of nuisance signals were then removed from the data through linear regression (white matter (WM), cerebrospinal fluid (CSF), and six motion parameters). Finally, fMRI data were temporally filtered with a band pass of 0.01-0.08 Hz. Recent studies have demonstrated that head motion has a substantial impact on FC [47, 48]. Thus, any subjects that exhibited translation in any of the cardinal directions larger than 1.5 mm or a maximum rotation larger than 1.5 degrees were excluded from the study. In addition, framewise displacement (FD) was evaluated in the three groups as suggested by Power et al. [47]. Finally, structural images were processed using the SPM8 toolbox. Spatial normalization to the MNI space was performed using a diffeomorphic anatomical registration through exponentiated Lie algebra and segmented into gray matter (GM), WM, and CSF. The segmented GM was modulated using nonlinear deformation. The total GM volume was then calculated. GM was entered as a global variable to correct for the global GM volume of different subjects in the statistical analysis.

### 2.5. Functional Connectivity Mapping

The regions of interest (ROIs) for the bilateral insular subregions were selected for the three groups. In previous literatures, three subregions of the insula were subdivided based on the clustering of FC patterns in unilateral insula [49, 50]. According to the template of the insula from the findings of Deen et al. [51], each of the three subregions was used as a seed to perform whole-brain FC analysis in the three groups. To perform FC analysis, the mean BOLD time series were extracted from these seeds. Subsequently, FC analysis was performed between the seeds and all voxels in the brain. The resulting correlation coefficients were transformed to approximate a Gaussian distribution using Fisher's *r*-to-*z* transformation.

### 2.6. Statistical Analysis

#### 2.6.1. Statistical Analysis of Participant Demographic Information

Age, years of education, and head motions (FD value) of the three groups were compared using one-way analysis of variance (ANOVA). A chi-squared test was used to compare gender distributions.

#### 2.6.2. Statistical Analysis of Functional Connectivity in Three Groups

After tests of normality, homogeneity of variance, and Mauchly's test for sphericity, one-way ANOVA and post hoc analyses were performed to determine differences in FC within each insular subregion with age, gender, years of education, and GM as covariates. First, the within-group *Z*-value map was analysed using a random effects one-sample *t*-test. Statistical maps of the significant connections of each seed were created for each group (*p* < 0.05, false discovery rate corrected, cluster size large than 23 voxels [52]). Second, we restricted the one-way ANOVA. To make sure the compared regions were significantly connected with the subinsular seed at least in one group, we created the mask from the related union of one-sample *t*-test results from three groups. The significance threshold of group differences for the ANOVA was set to *p* < 0.01 with a minimum cluster size of 23 voxels for corrected significance at *p* < 0.05. We extracted the average *z*-score of the regions that showed significant changes in the ANOVA in three groups. Then, post hoc analyses were performed on the mean *z*-scores of each seed to identify any differences.

#### 2.6.3. Relationship between Psychological Scores and Functional Connectivity

In dancer and musician groups, we first calculated the correlation between C-IRI total score and the FC (*z*-score) within six insular subnetworks, respectively (*p* < 0.05, cluster size large than 23 voxels). Because the distribution of the C-IRI score was a ranked distribution, we used spearman correlation analysis in this study with covariance of age, gender, education years, and GM. Second, we measured the relationship between C-IRI scores and the changed FC (*z*-score) in dancer and musician groups, respectively. Moreover, we assessed the difference of these relationships between the two groups using the permutation test.

## 3. Results

### 3.1. Participant Demographic Information

Based on our exclusion criterion (head motion), we eliminated 5 subjects (2 dancers, 3 musicians) and had 65 participants (21 dancers, 20 musicians, and 24 controls) in total. Demographic information is presented in Table 1. The three groups did not differ significantly in gender, age, FD, or years of education (see Table 1).

### 3.2. Differences in Psychological Scores

The mean scores of the C-IRI in the dancer and musician groups were higher than the reference scores from the large sample (*n* = 529) (see Table 2) [46]. The dancers and musicians had higher mean scores in the C-IRI total and three subscales compared with the large sample. In addition, the dancer and musician groups had C-IRI total, PT, and PD scores as high as one standard deviation above the large sample. There was no significant difference in the C-IRI between the dancers and musicians.

### 3.3. Portion of the Results Part.y

Through one-way ANOVA, we found that there were different group effects in the subregions of the insula with cognition, emotion, and motor networks (see Table 3). The specific findings were described as below.

**Figure 1:**
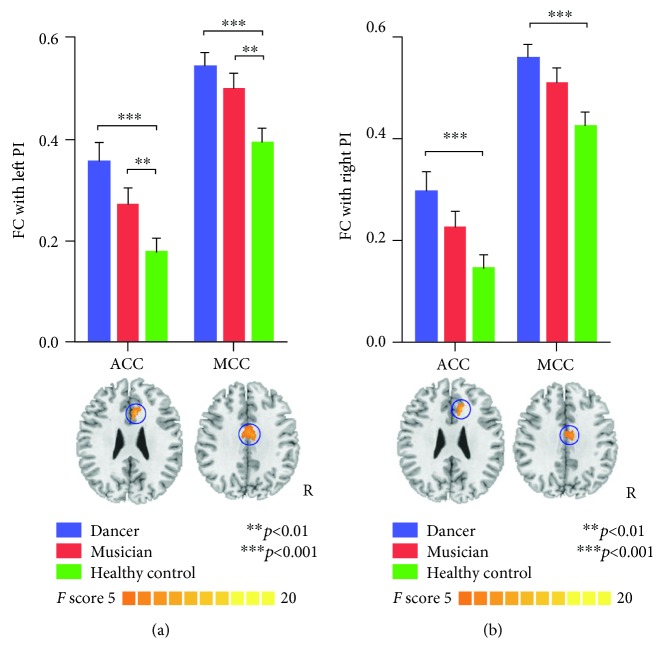
Functional connectivity of PI seeds. (a) Represents the results of the left PI, and (b) represents the results of the right PI. There is increased FC between PI and ACC and MCC in dancers and musicians compared with controls.

PI: one-way ANOVA of the correlations between PI and other brain regions showed significant differences in the anterior cingulated cortex (ACC) and middle cingulated cortex (MCC), with significantly increased FC between the left PI and ACC and MCC in training groups compared with the control group, as well as increased FC between the right PI and ACC and MCC only in the dancer group (see Table 3, Figure 1).

**Figure 2:**
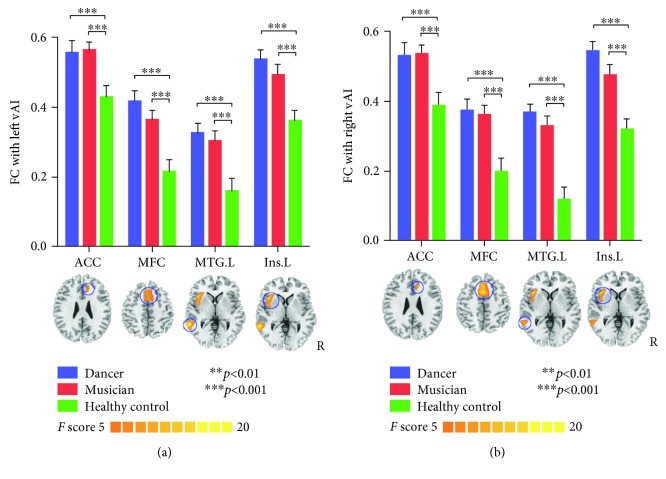
Functional connectivity of vAI seeds. (a) Represents the results of left vAI, and (b) represents the result of the right vAI. Increased FC is observed in ACC, MFC, left MTG, and left insula in dancers and musicians compared with controls.

vAI: one-way ANOVA of the correlations between vAI and other brain regions showed significant differences in ACC, the medial frontal cortex (MFC), middle temporal gyrus (MTG), and left insula. The post hoc analysis results revealed that increased FC was observed in musician and dancer groups compared with the control group (see Table 3, Figure 2).

**Figure 3:**
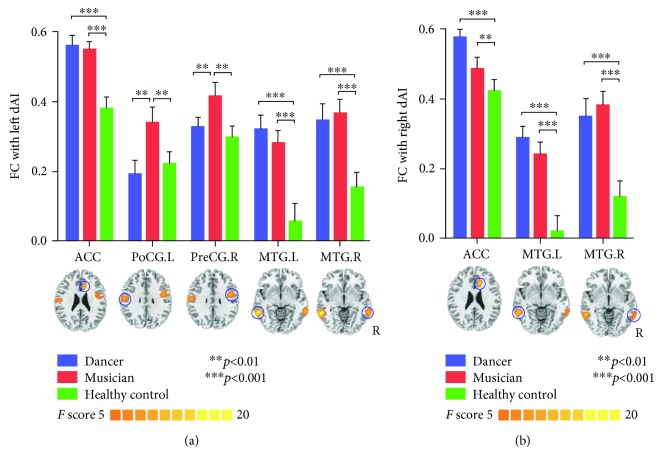
Functional connectivity of dAI seeds. (a) Represents the results of the left dAI, and (b) represents the results of the right dAI. A significant increase in FC between dAI and ACC and left MTG is observed in dancers and musicians compared with controls. Only in the musician group is there a significantly higher FC between dAI and left postcentral and right precentral gyrus.

dAI: one-way ANOVA of the correlations between dAI and other brain regions showed significant differences in ACC, left MTG, left postcentral, and right precentral gyrus. After post hoc analysis, significantly increased correlations between dAI and ACC and left MTG and right MTG were observed in training groups compared with controls. Furthermore, we found significantly higher FC between the left dAI and the left postcentral gyrus and right precentral gyrus only in musicians compared with dancers and controls. There were no such correlations between dancers and controls (see Table 3, Figure 3).

### 3.4. The Relationship between FC and C-IRI Scores

The relationships were found between C-IRI total score and FC within six insular-subregion networks in the dancer and musician groups, respectively. In the dancer group (see SFigure 1), within the dAI network the associations were located in the posterior insula, MCC, and inferior frontal gyrus (IFG). Within the PI network, high relationships were found in the precentral gyrus, postcentral gyrus, and supplementary motor area (SMA). Within the vAI network, the high correlations were shown in the temporoparietal junction (TPJ), ACC, and IFG. In the musician group (see SFigure 2), we found high relationships in the basal ganglia, SMA, and precentral gyrus within dAI and vAI networks. Within the PI network, the high correlations were located in the pre-SMA and precentral gyrus.

**Figure 4:**
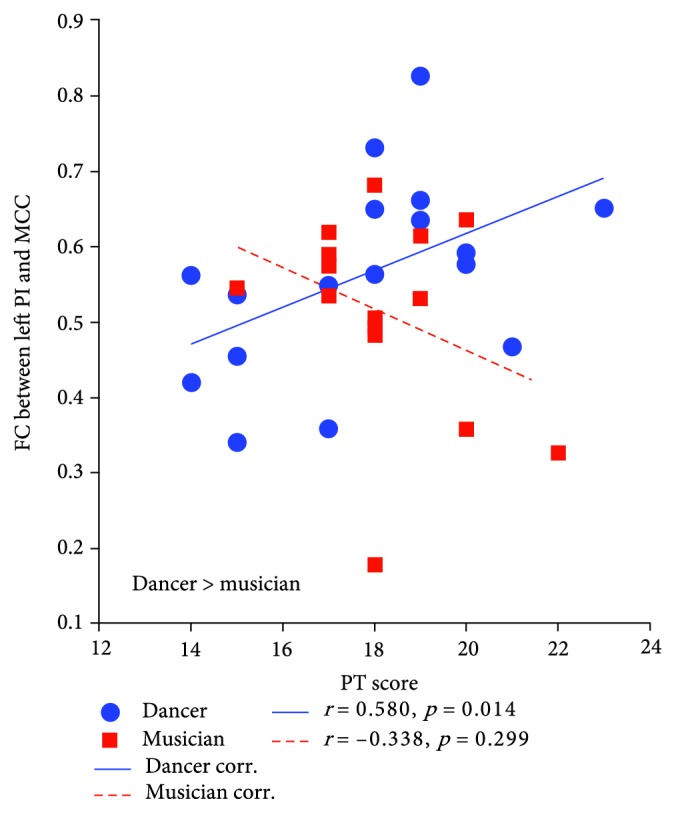
The positive associations between C-IRI scores and FC in the dancer group. Linear partial correlation coefficients between FC (left PI and MCC) and PT scores in dancers (blue solid line) and musicians (red broken line).

**Figure 5:**
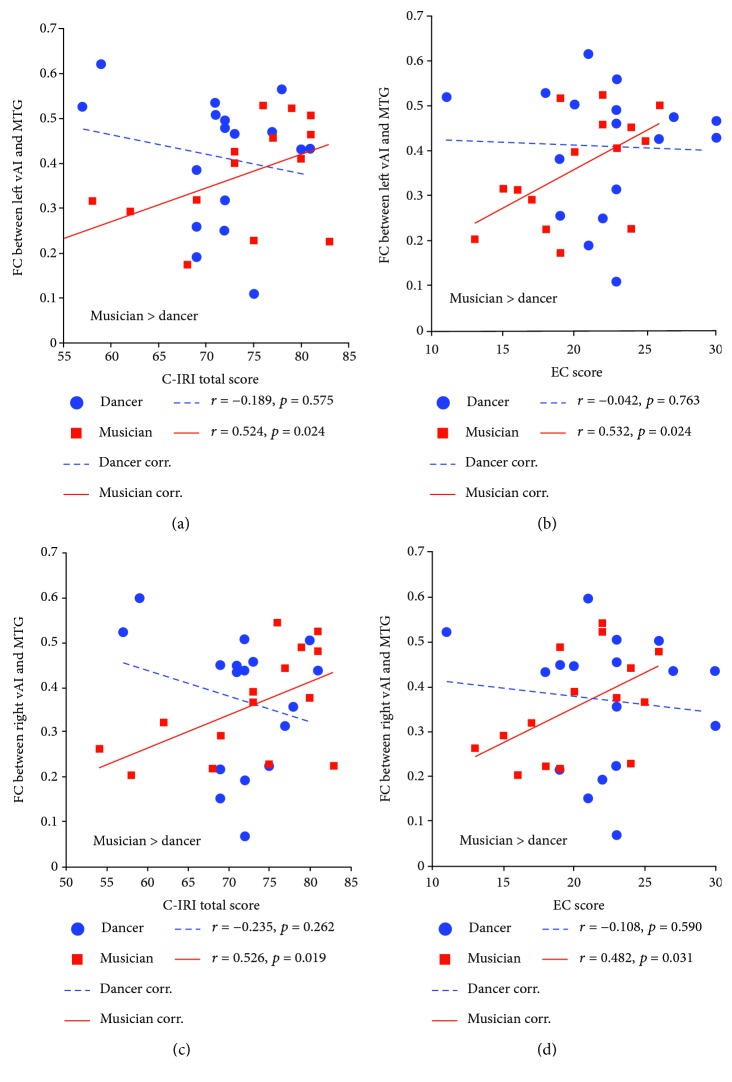
The positive associations between C-IRI scores and FC in the musician group. Linear partial correlation coefficients between FC and C-IRI scores in dancers (blue broken line) and musicians (red solid line). (a) Shows correlations between FC (between left vAI and MTG) and C-IRI total scores. (b) Shows correlations between FC (between left vAI and MTG) and EC scores. (c) Shows correlations between FC (between right vAI and MTG) and C-IRI total scores. (d) Shows correlations between FC (between right vAI and MTG) and EC scores.

Compared with musicians, the dancers showed significant positive associations between FC of PI-MCC and PT scores (see Figure 4, Table 4: *r* = 0.580, *p* = 0.014; dancer > musician). In the musician group, positive associations were observed between EC and FC (see Figure 5, Table 4: left vAI and MTG: *r* = 0.532, *p* = 0.024; right vAI and MTG: *r* = 0.482, *p* = 0.031, musician > dancer). The same associations were found between total scores and FC (see Figure 5, Table 4: left vAI and MTG: *r* = 0.524, *p* = 0.024; right vAI and MTG: *r* = 0.526, *p* = 0.019, musician > dancer).

## 4. Discussion

As far as we know, this study was the first to identify the dance/music training effects on insular circuit and associated the effects with empathic ability. The overall connectivity pattern of insular subdivisions conformed to the former studies, including the posterior, dorsal anterior, and ventral anterior portions [33]. As hypothesized, the dancer and musician groups indicated stronger insular connectivity recruited in the empathic process, including interoceptive sensorimotor representation (the PI subnetwork), affective evaluation (the vAI subnetwork), and higher-level cognitive control (the dAI subnetwork). Moreover, we found different insular FC patterns related to empathy scores between dancers and musicians. In brief, the changed functional connectivity within insular networks found in training groups partly overlapped with the empathic circuit. Those results might further explain the association between enhanced insular FC and empathic ability scores.

### 4.1. Enhanced Insular Subnetwork Connectivity with Shared Representations

The shared representations between self and others are key components of empathy, which rely on the neural coding of a somatosensory and sensorimotor process in action perception and performance [29]. The shared presentations help to judge and feel the emotional states of others. The PI integrates somatosensory signals from limbic structures [53] and forms the primary interoceptive representations of visceral and sensory stimuli from the body state (linearly related to objective stimulus intensity) [54]. Specifically, the PI together with MCC forms a representation of pain and internal bodily states [32, 55]. It is very important for empathy for pain, for these representations are further processed by AI and form subjective feelings. Our findings of the enhanced PI subnetwork indicated a better representation ability in the dancer/musician facilitating for further empathic processes. Besides, the enhanced FC between PI and ACC indicated a high participation in the salience process. The increased FC of PI within the salience network in the dancer/musician group might promote the integration between the interoceptive and exteroceptive stimulus, which helped to improve the function between the sensorimotor process and the response to external stimulus. Therefore, the PI subnetwork usually grounds subjective feelings [56] and might further affect the empathic feeling about others. Dysfunctional connectivity of PI has a negative influence on interoceptive awareness of emotion in autism [57]. Taken together, the intensive sensorimotor training in dance/music might promote the PI subnetwork function, which further facilitates the effective representation process in vAI. The vAI is functionally in charge of further processing of visceral-autonomic information afferents from PI, and it is of crucial importance for representing the emotional experience [58]. It might be a link between the mirror neuron system and emotional processing, which is essential to empathy [59] and is a neuro-foundation for dance therapy [60] or dance expression [61]. The vAI together with ACC is the core structure in empathy [62], and it is specialized for the anticipation and evaluation of emotional stimuli [63]. The MTG, which is a part of the magnocellular pathway, is thought to enroll in processing the emotional facial expressions [64]. The MFC zone is associated with negative affect and reward and processes low-level affective signals into cognitive control [65]. Our findings showed an improved vAI subnetwork function with effective process and representation. Therefore, the results suggested a better sensorimotor information integration in the PI subnetwork and promotion of affective function in the vAI subnetwork in dancers/musicians. Prolonged dance/music training might strengthen the somatosensory and sensorimotor representation through action learning and executing.

### 4.2. Enhanced dAI Subnetwork Connectivity with Self-Other Awareness

Self-other awareness is a vital component of human empathy, which helps us to understand ourselves and others [29]. The dAI subnetwork plays a critical role in the sense of self-agency and helps to distinguish self from the others. The structure is related to high-level cognitive processes and is consistently activated across all task domains compared with other insular subdivisions. It functions as a hub to integrate information from PI and vAI and dynamically coordinates key nodes of other large-scale brain networks (the CEN and the DMN) [66, 67]. The dAI and ACC are the core structures of the salience network which integrates external stimuli with internal homeostatic contexts and detects salience events [68]. The dAI subnetwork is proposed to integrate images of one's own feelings, the sensory environment, and the motivational, hedonic, and social interactions between these into one whole representation of the sentient self [32]. Dance/music training is a multisensory art form requiring the trainees to process and integrate the external/internal stimuli and result in an enhanced salience integration with the dAI subnetwork. This improved salience function was a neuro foundation and facilitate to form a sense of self. There are anomalous functional connectivity patterns with dAI networks in autism and schizophrenia which have a deficit in self-other awareness [69, 70]. Our study found significantly increased FC between dAI and ACC and MTG in dancer and musician groups. It suggested an efficient salience process and the agency of self in dAI.

As mentioned above, the insula is a vital structure in saliency processing and facilitates the generation of subjective feelings and emotions from the integration of interoceptive and exteroceptive sensory information. The three insular subnetworks collaborate as a whole [71]. Any change of any subnetwork can affect the whole insular network. In this study, the improved PI subnetwork function promoted the vAI subnetwork. The improved PI and vAI function finally affected the dAI function and might result in a high integration of agency of self. These changes ultimately optimize the processing pattern of the whole insula network. Therefore, we inferred that the PI and vAI functions might very important for higher emotion and cognition. Through multi-sensorimotor action training in dance/music, the representation of self and others had been improved and finally affected the awareness of self and others, which result in changed empathic ability ultimately.

### 4.3. The Discrepant Insular FC Patterns Related to Empathy Score

There were discrepant insular FC models related to empathic ability between dancer and musician groups. The robust evidence has indicated the empathy-related activations in salience and sensorimotor functional regions, including AI, ACC, and primary sensorimotor cortex [30, 72]. Recently, relevant studies also reported the similar findings that the sensorimotor-insula-frontal loop overlaps the empathic networks [73, 74]. The insula acts as a mediator to enhance the information process in the sensorimotor-insula-frontal loop and improve the interaction between the primary sensorimotor cortex and higher-order regions [73]. Therefore, the empathic ability partly might rely on sensorimotor systems that underpin our feelings of bodily state [75]. Compared with musicians, we found that the empathy index (C-IRI-total score) was significantly correlated with the dAI, vAI, and PI functional networks in dancers. Within the vAI network, the empathy index was mainly connected with TPJ which was the central hub to process multimodal sensory representations [76] and had been proposed as the main component in the salience network [31]. Within the dAI network, the empathy index was connected with the IFG which is a part of MNS [77]. It is related not only to motor actions but also to emotion, recognition, and empathy [39, 78]. Within the PI network, pre/postcentral gyrus and SMA were mainly correlated with the empathy index. Moreover, compared with the musician group, the increased relationship was observed between the PT score and FC of PI-MCC. Thus, high coupling was found between empathy measurements and the sensorimotor-insula-frontal functional loop in the dancer group. The dance training emphasized embodied recognition and action imitation with whole body movement execution [75]. It might improve the integration of the primary sensorimotor process and affect the cognitive domain of empathy in dancers.

Within empathic circuits, the higher-order areas interact with regions encoding the bottom-up-driven affective representations and response, especially with the anterior insula and the basal ganglia [79]. Compared with the primary sensorimotor cortex, the basal ganglia has a higher function. It affects planning for movement and attends to control metric elementary movement [80]. As mentioned earlier, the SMA is the core structure of empathy [40]. It is in charge of movement programming and forms a queue of sequenced motor commands before voluntary movements are executed by the primary motor area [81]. The insula acts as a mediator to couple the motor controlling regions and frontal areas. In our study, we found that the C-IRI-total score was significantly correlated with the dAI, vAI, and PI functional networks in the musician group. Within three function networks, there were correlations with SMA. Within the dAI and vAI networks, there were correlations with the basal ganglia. Moreover, compared with the dancer group, the increased relationship was observed between EC and total score and FC of vAI-MTG in musicians. Enhanced FC was found between dAI and the sensorimotor cortex only in musicians. Therefore, there was efficient coupling in the motor control-insular-frontal functional loop in musicians. The music training is an audio-visual-motor feedback process which needs a quick feedback and dedicated control of matric movements. It improved the encoding of higher motor controlling which might affect the emotional domain of empathy in musicians.

## 5. Limitation

Our research attributed the insular subnetwork functional differences observed between groups to brain plasticity related to their specific training. However, our research was a cross-sectional design which could not exclude the possible differences preexisting in functional connectivity between groups. Longitudinal studies of dance/music training are recommended to verify whether functional brain plasticity is due to training or preexisting differences.

In this paper, the data of the control group was obtained from the database of the UESTC Imaging Centre without C-IRI scores. To make a comparison, we looked at differences in empathic scores of the expert sample in contrast to a large Chinese sample from another study. However, this is particularly defective as the contrasts were against unequal sample sizes. The observed differences might not fully link differences in FC between dancers, musicians, and controls to differences in empathic abilities.

Apart from the insular network, there are other brain areas and structures vital in the empathic process. At present, our research had only focused on insular function. The validity of evidence with the link between insular network function and empathic ability is limited. However, our findings did indicate the enhanced insular FC in the dancer/musician group and the insula was an important structure in the empathic network.

## 6. Conclusion

This study investigated the insular functional connectivity related to empathy in the dancer/musician group. The findings indicated that the dance/music training induced enhanced insular subnetwork functions which were associated with improved empathic ability. The insular network integrated the external/internal information and acted as the basic neural foundation for higher functions. Increased insular function induced by expert training could result in an improved fundamental information processing and facilitated other high-level ability. Our results gave a neuroplasticity perspective to the understanding of the transfer effects of long-term training and provided constructive guidance for dance or music therapy.

## Figures and Tables

**Table 1:** Participant demographic information.

	Dancer group	Musician group	Control group	*p*
Gender (male/female)	7/14	7/13	7/17	0.91^a^
Age (mean ± SD years)	18.71 ± 1.90	19.05 ± 1.19	19.58 ± 1.58	0.19^b^
Education (years)	12.48 ± 1.63	12.50 ± 1.19	13.03 ± 1.42	0.21^b^
FD	0.1021 ± 0.0492	0.0850 ± 0.0493	0.0919 ± 0.0328	0.5972^b^

Indicated values shown are mean ± standard deviation. ^a^The *p* values according to a chi-squared test between the dance, music, and control groups. ^b^The *p* values according to the ANOVA test. FD indicates framewise displacement.

**Table 2:** Scores of C-IRI in dancer and musician groups.

	Dancer group	Musician group	*p*
C-IRI total	63.47 ± 6.23	64.13 ± 8.24	0.79
PT	17.76 ± 2.56	18.20 ± 1.66	0.58
FS	12.59 ± 2.92	14.07 ± 3.73	0.22
EC	19.29 ± 4.57	17.20 ± 3.91	0.18
PD	13.82 ± 3.30	14.67 ± 3.58	0.49

Indicated values shown are mean ± standad deviation.

**Table 3:** Insular subdivision connectivity in dancers, musicians, and controls.

Region	BA	MNI coordinates			Peak *F* score	Cluster voxels	Post hoc analysis		
		*x*	*y*	*z*			a (dancer-control)	b (musician-control)	c (dancer-musician)
Seed: left PI									
ACC	BA 24	5	26	26	8.95	36	*p* < 0.001	*p* = 0.038	—
MCC	BA 23	9	-12	39	8.87	32	*p* < 0.001	*p* = 0.019	—
Seed: right PI									
MCC	BA 48	6	-19	40	9.21	68	*p* < 0.001	—	—
ACC	BA 48	6	24	26	9.32	39	*p* < 0.001	—	—
Seed: left vAI									
MFC	BA 8	-4	19	51	14.23	246	*p* < 0.001	*p* = 0.001	—
INS.L	BA 48	-38	15	2	13.32	162	*p* < 0.001	*p* = 0.005	—
MTG.L	BA 21	-63	-48	-2	8.86	85	*p* = 0.001	*p* = 0.003	—
ACC	BA 32	10	29	33	8.58	46	*p* = 0.012	*p* = 0.002	—
Seed: right vAI									
MFC	BA 8	-3	23	51	12.65	194	*p* = 0.002	*p* = 0.002	—
INS.L	BA 48	-36	16	2	15.58	171	*p* < 0.001	*p* < 0.001	—
MTG.L	BA 21	-61	-47	-2	20.55	127	*p* < 0.001	*p* < 0.001	—
ACC	BA 32	13	29	23	7.96	42	*p* = 0.010	*p* = 0.003	—
Seed: left dAI									
MTG.L	BA 21	-61	-47	-4	13.23	124	*p* < 0.001	*p* = 0.001	—
MTG.R	BA 21	61	-44	-6	9.63	108	—	*p* = 0.001	—
ACC	BA 32	10	29	24	14.48	96	*p* < 0.001	*p* = 0.003	—
Seed: right dAI									
MTG.L	BA 21	-63	-48	-4	19.09	168	*p* < 0.001	*p* < 0.001	—
ACC	BA 32	10	31	25	13.28	102	*p* < 0.001	—	*p* = 0.032
PoCG.L	BA 43	-59	-14	31	11.09	122	—	*p* = 0.002	*p* = 0.001
MTG.R	BA 21	62	-41	-6	11.01	127	—	*p* < 0.001	—
PrCG.R	BA 6	46	2	31	10.07	119	—	*p* = 0.003	*p* = 0.002

a indicates the values for post hoc analysis between the dancers and the controls. b indicates the values for post hoc analysis between the musicians and the controls. c indicates the values for post hoc analysis between the dancers and the musicians. — indicates that the comparison results of post hoc analysis were not significant.

**Table 4:** Relationship between FC and C-IRI scores.

C-IRI scores	Total			PT			EC		
	D (*r*/*p*)	M (*r*/*p*)	D-M	D (*r*/*p*)	M (*r*/*p*)	D-M	D (*r*/*p*)	M (*r*/*p*)	D-M
FC (left PI-MCC)	-0.353/0.264	-0.247/0.182	0.473	0.580/0.014^∗^	-0.338/0.299	0.004^∗^	0.261/0.271	0.249/0.295	0.912
FC (left vAI-MTG)	-0.189/0.575	0.524/0.024^∗^	0.036^∗^	0.281/0.273	0.264/0.341	0.426	-0.042/0.763	0.532/0.024^∗^	0.022^∗^
FC (right vAI-MTG)	-0.235/0.262	0.526/0.019^∗^	0.013^∗^	-0.042/0.687	0.317/0.242	0.642	-0.108/0.590	0.482/0.031^∗^	0.017^∗^

D represents dancer group; M represents musician group.

## Data Availability

The fMRI data used to support the findings of this study are available from the corresponding author upon request.

## Supplementary Materials

Supplementary Materials The correlation between C-IRI score and FC within six insular subregion networks in the dancer/musician group.
