# Supplementary material for: Increased Insular Connectivity and Enhanced Empathic Ability Associated with Dance/Music Training
Source: Neural Plast. 2019 May 6;2019:9693109. doi: 10.1155/2019/9693109 (PMC6526550; doi:10.1155/2019/9693109)
Supplement: Supplementary Materials — The correlation between C-IRI score and FC within six insular subregion networks in the dancer/musician group. [file 9693109.f1.pdf]

Supplementary material

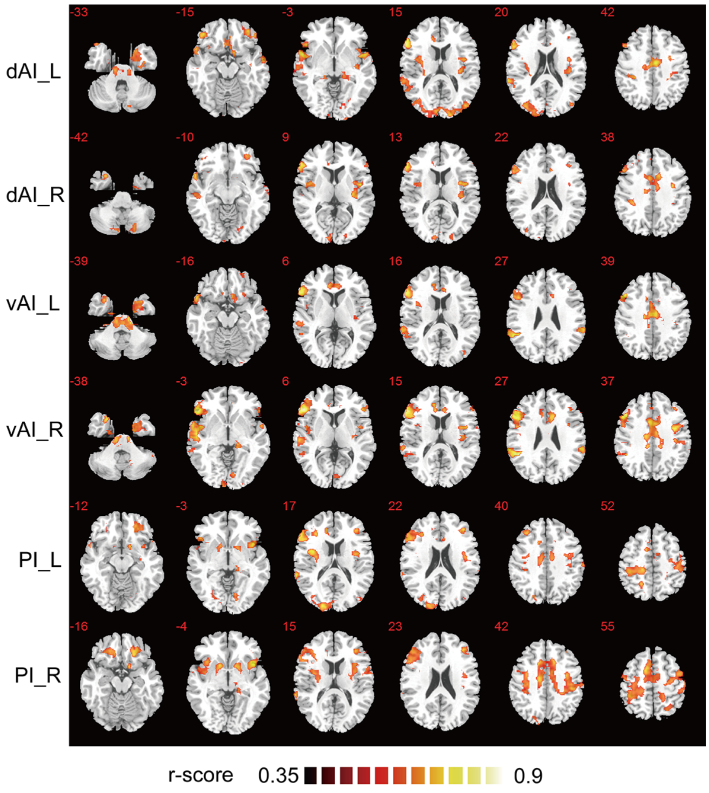

SFigure1: The correlation coefficients were shown between C-IRI total score and FCs within six insular sub-region networks in dancer group.

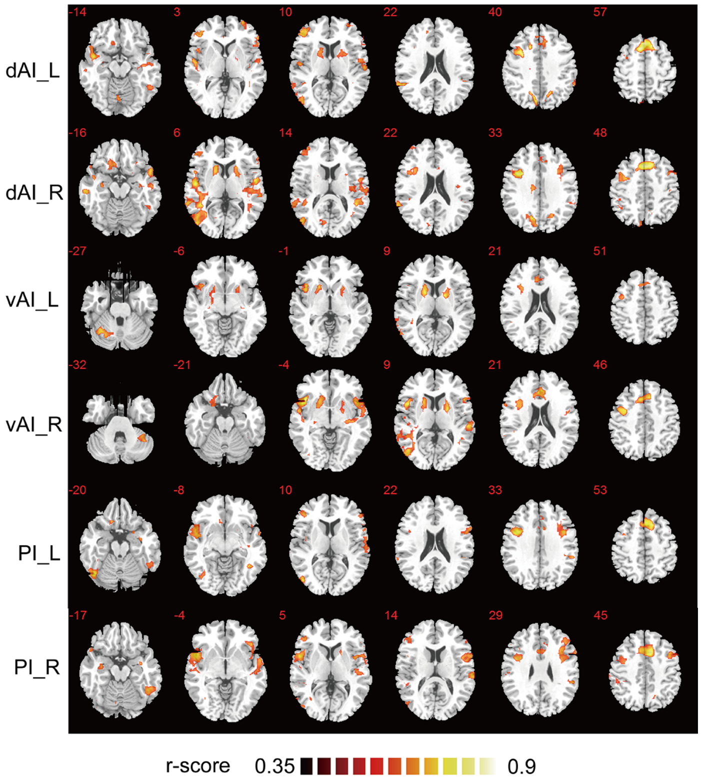

Figure 2: The correlation coefficients were shown between C-IRI total score and FCs within six insular sub-region networks in musician group.
